# Proteinuria is independently associated with carotid atherosclerosis: a multicentric study

**DOI:** 10.1186/s12872-021-02367-x

**Published:** 2021-11-19

**Authors:** Wencai Jiang, Meixiang Chen, Jianyu Huang, Yu Shang, Changyu Qin, Zheng Ruan, Shuang Li, Ruixin Wang, Pengfei Li, Yuekang Huang, Jianxiong Liu, Lin Xu

**Affiliations:** 1 Department of Cardiology, Chengdu Second People’s Hospital, Chengdu, China; 2 The First Affiliated Hospital of Guangdong Pharmaceutical University, Guangzhou, China; 3 Department of Geratic Cardiology, General Hospital of the Southern Theatre Command, Guangzhou, China; 4 The First School of Clinical Medicine, Southern Medical University, Guangzhou, China; 5 School of Automation, Guangdong University of Technology, Guangzhou, China; 6 The People’s Hospital of Wenchuan, Wenchuan, China

**Keywords:** Atherosclerosis, Proteinuria, Cardiovascular diseases, Risk factors, Epidemiology

## Abstract

**Background and aims:**

Atherosclerosis is a vital cause of cardiovascular diseases. The correlation between proteinuria and atherosclerosis, however, has not been confirmed. This study aimed to assess whether there is a relationship between proteinuria and atherosclerosis.

**Methods:**

From January 2016 to September 2020, 13,545 asymptomatic subjects from four centres in southern China underwent dipstick proteinuria testing and carotid atherosclerosis examination. Data on demography and past medical history were collected, and laboratory examinations were performed. The samples consisted of 7405 subjects (4875 males and 2530 females), excluding subjects failing to reach predefined standards and containing enough information. A multivariate logistic regression model was used to adjust the influence of traditional risk factors for atherosclerosis on the results.

**Results:**

Compared with proteinuria-negative subjects, proteinuria-positive subjects had a higher prevalence rate of carotid atherosclerosis. The differences were statistically significant (22.6% *vs.* 26.7%, χ^2^ = 10.03, *p* = 0.002). After adjusting for common risk factors for atherosclerosis, age, sex, BMI, blood lipids, blood pressure, renal function, hypertensive disease, diabetes mellitus and hyperlipidaemia, proteinuria was an independent risk factor for atherosclerosis (OR = 1.191, 95% CI 1.015–1.398, *p* = 0.033). The Hosmer–Lemeshow test was used to test the risk prediction model of atherosclerosis, and the results showed that the model has high goodness of fit and strong independent variable prediction ability.

**Conclusions:**

Proteinuria is independently related to carotid atherosclerosis. With the increase in proteinuria level, the risk of carotid atherosclerotic plaque increases. For patients with positive proteinuria, further examination of atherosclerosis should not be ignored.

## Introduction

Cardiovascular diseases (CVDs) are the leading cause of human deaths globally [1]. Not merely in high-income countries, the number of patients suffering from CVD has surged in low- and mid-income countries [2]. It is estimated that 23.6 million people will die of CVD every year by 2030 [3]. CVD brings increasing burdens to individuals, families and health care systems. Atherosclerosis is a nosogenesis of CVD. Atherosclerotic plaque rupture is closely related to cardiovascular events. Identifying atherosclerosis and restricting plaque progression can help reduce the likelihood of plaque rupture and myocardial infarction [4].

Recently, some studies have found that proteinuria is associated with diabetes mellitus and CVD all-cause mortality [5–7]. Microalbuminuria has been proven to be associated with all-cause mortality in developed countries [8, 9]. Therefore, simple and noninvasive dipstick proteinuria testing may potentially reveal atherosclerosis. Approximately 80% of global deaths caused by CVD occur in mid- and low-income countries. Nearly 4 billion individuals lived in moderate poverty with a daily income of fewer than 5 dollars. Dipstick proteinuria testing is cost-effective, especially for developing countries without sufficient health resources [10].

The correlation between proteinuria and carotid atherosclerosis is not clear. Previous studies focused on the correlation between proteinuria and death in terms of mortality, showing that there is still a disparity in mortality after correcting for hypertension and diabetes mellitus [11–13]. However, these studies have not mentioned atherosclerosis; some authors even deny the correlation between proteinuria and carotid atherosclerosis [14]. In addition, the relationship between proteinuria and carotid atherosclerosis has not been confirmed in enough samples. The study aimed to determine the correlation between atherosclerosis and proteinuria values in routine urine tests via dipstick proteinuria testing, identify a new reliable predictor to screen out atherosclerosis against death caused by CVD, and establish a new risk prediction model of atherosclerosis.

## Methods

**Fig. 1:**
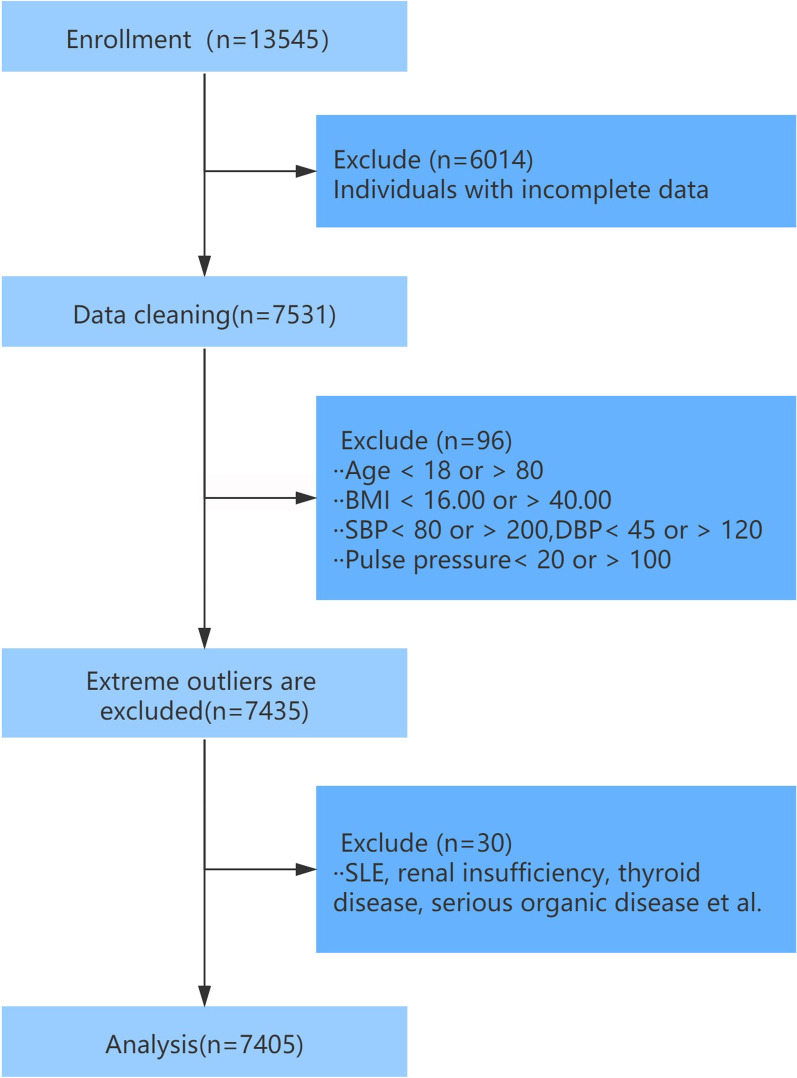
The flow of participants from screening

The subjects were 13,454 asymptomatic populations taking dipstick proteinuria testing and carotid atherosclerosis in four of the top three large-scale hospitals (The General Hospital of the Southern Theatre Command, PLA, the First Affiliated Hospital of Guangdong Pharmaceutical University, the Subei People’s Hospital and the First Affiliated Hospital of Chengdu Medical College) in China from January 2016 to September 2020. Subjects with the following conditions were excluded: (1) a lack of valid information; (2) age < 18 or > 80 years; (3) BMI value < 16.00 or > 40.00 kg/m^2^; (4) systolic blood pressure < 80 or > 200 mmHg, diastolic blood pressure < 45 or > 120 mmHg and pulse pressure < 20 or > 100 mmHg; (5) subjects with systemic lupus erythematosus, thyroid disease, autoimmune disease, nephritis, nephrotic syndrome, renal insufficiency(eGFR < 60 ml/min/1.73 m^2^, the eGFR value was calculated according to the MDRD formula), congestive heart failure, malignant tumour, severe organic disease. The total number of subjects was 7405, including 4875 males and 2530 females. The procedure of subject selection process is shown in Fig. 1.

### Atherosclerosis testing

The carotid atherosclerosis examination of the subjects’ common carotid arteries, carotid sinus, and carotid internal and external arteries was performed by senior sonographers with an HA-500 colour ultrasound instrument (HITACHI, Japan). According to the recommendations of the American Society of Echocardiography Recommendation, if CIMT was under 0.8 mm without plaques, the carotid artery was proven to be normal; if CIMT was above or equal to 0.8 mm without plaques, CIMT was considered to be increased, and plaque was considered a focal thickening that encroached into the lumen by 0.5 mm or by 50% of the surrounding intimal-medial thickness or where CIMT was > 1.5 mm [15]. Each subject was tested two times, and the data were taken as an average [16].

### Proteinuria testing

Professional laboratory physicians used AUTION Sticks 10EA urine analysis paper (ARKRAY, Japan) to analyse fresh urine samples and then used an AUTION AX-4030 urine analyser (ARKRAY, Japan) to interpret the test strip at 60 s. The results of dipstick proteinuria testing were grouped by negative < 15 mg/dL, “ ± ” ≥ 15 mg/dL, “1 + ” ≥ 30 mg/dL, “2 + ” ≥ 100 mg/dL, “3 + ” ≥ 300 mg/dL and “4 + ” ≥ 1000 mg/dL. The “ negative” was considered to be without proteinuria, “ ± ” and “ 1 + ” mild proteinuria, “ 2 + ”, “ 3 + ” and “ 4 + ” severe proteinuria. To avoid the influence of eating and drinking, we selected the morning urine of the subjects to detect proteinuria.

### Laboratory examination

Fasting blood samples were taken from each subject’s antecubital vein by professional nursing staff; blood lipids, blood glucose and creatinine were measured by professional clinical physicians via Cobas c 702 AEROSET (Roche, Germany). If TGs were above 2.30 mmol/L, total cholesterol was above 6.20 mmol/L, LDL was above 4.10 mmol/L or HDL was under 1.00 mmol/L, the subject was considered to have hyperlipemia. If fasting blood glucose was above 7.0 mmol/L, a subject took hypoglycaemic agents or had a history of diabetes mellitus, he was considered to have diabetes mellitus [17].

### Collection of other clinical data

The height and weight without shoes and with light clothes of each subject was measured by trained professional medical staff. The BMI value equaled the weight value (kg) divided by the square root of the height value (m). The blood pressure of each subject was measured by trained medical staff via an electronic sphygmomanometer. Before testing, each subject rested for at least 15 min in a chair on their backs. The measurements were taken three times, and the data were taken as an average. The pulse pressure equaled the SBP minus the DBP. If the SBP was above 130 mmHg or the DBP was above 80 mmHg, the subject took hypertensive drugs or had a history of hypertension, he was considered to have hypertension [18].

### Statistical analysis

Categorical variables are represented by percentages (%), the comparison of which was performed by the Chi-square test. Quantitative variables are represented by the mean value ± standard deviation, the comparison analysis of which was performed by the independent sample Student’s t test. The analysis of the correlation between proteinuria and carotid atherosclerosis was evaluated by the Chi-square test. The multivariate logistic regression model was used to estimate the adjusted OR. A cross-sectional study of a simple survey group was carried out to confirm the correlation between proteinuria and carotid atherosclerosis. All statistical analyses were performed using SPSS 26.0 (IBM, U.S.). If *p* < 0.05, the difference was statistically significant.

## Results

**Fig. 2:**
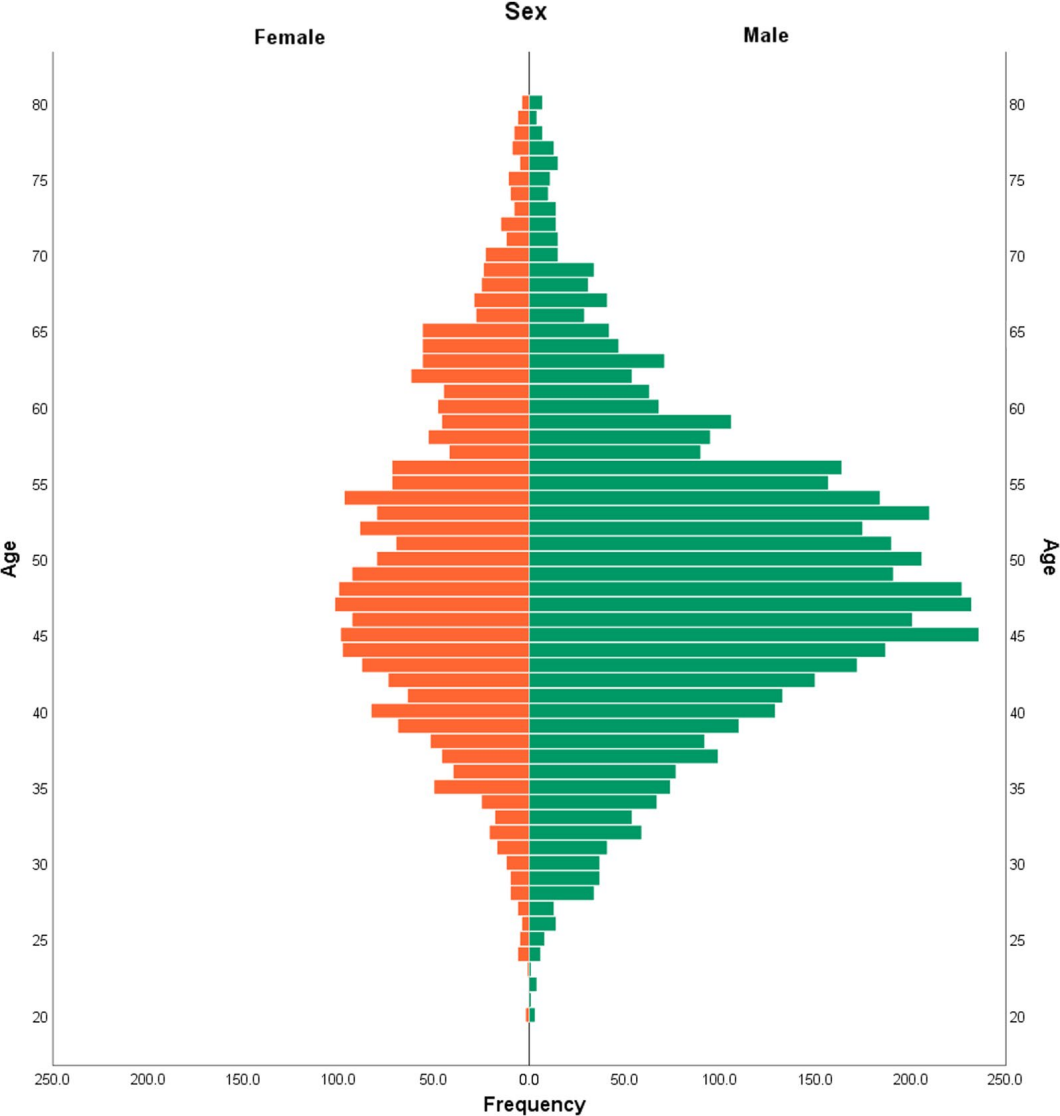
The age distribution of men and women

The original subjects were 13,545 patients from four centres. Of them, 7,405 with adequate baseline data were defined as the sample. They were aged 18–80 years (average age: 49.15 ± 10.24 years) and included 4875 males (65.8%) and 2530 females (34.2%). The age distribution of men and women is shown in Fig. 2. Most of the female subjects were 35–65 years old, whereas the male subjects were 35–60 years old. The overall age of the female subjects was older than that of the male subjects. The prevalence of carotid atherosclerotic plaque in males was higher than that in females (26.4% *vs.* 17.2%, χ^2^ = 79.81, *p* < 0.05).

The proportion of proteinuria and carotid atherosclerosis in the population. A total of 1,215 subjects with proteinuria accounted for 16.4%, including 1,124 with mild proteinuria and 91 with severe proteinuria. According to carotid atherosclerosis in the carotid colour ultrasonography examination (CIMT ≥ 1.5 mm or the form of plaques), subjects were classified into two groups: the group without carotid atherosclerosis (n = 5684, accounting for 76.7%) and the group with carotid atherosclerosis (n = 1721, accounting for 23.2%). The group without carotid atherosclerosis was further classified into the subgroup with normal carotid atherosclerosis (n = 4768) and the CIMT subgroup (n = 916).

The comparison of baseline data between the carotid plaque group and the group without carotid plaque is shown in Table 1. The proteinuria rate, age, male proportion, BMI, SBP, DBP, pulse pressure, prevalence of hypertension, prevalence of diabetes, fasting blood glucose, triglyceride, total cholesterol, low-density lipoprotein and creatinine in the carotid plaque group were significantly higher than those in the group without carotid plaque, whereas high-density lipoprotein was lower than that in the group without carotid plaque.

**Table 1:** Baseline characteristics of the participants with and without carotid atherosclerosis

	Without carotid atherosclerosis (N = 5684)	With carotid plaque (N = 1721)	*p* value
Proteinuria, n (%)	888 (15.7%)	338 (18.9%)	0.002
Age (years)	47.0 ± 9.4	56.4 ± 9.6	0.000
Male, n (%)	3588 (63.1%)	1287 (74.8%)	0.000
BMI (kg/m^2^)	24.51 ± 3.29	24.96 ± 3.13	0.000
SBP (mmHg)	118.9 ± 15.8	128.8 ± 17.7	0.000
DBP (mmHg)	73.4 ± 11.4	71.3 ± 11.7	0.000
Pulse pressure (mmHg)	45.6 ± 9.9	51.5 ± 12.9	0.000
Hypertension, n (%)	760 (13.4%)	508 (29.5%)	0.000
Diabetes mellitus, n (%)	357 (6.3%)	254 (14.8%)	0.000
Hyperlipidaemia, n (%)	1839 (32.4%)	698 (40.6%)	0.000
FBG (mmol/L)	5.17 ± 1.25	5.75 ± 2.01	0.000
Triglycerides (mmol/L)	1.68 ± 1.43	1.88 ± 1.80	0.000
Total cholesterol (mmol/L)	5.00 ± 0.95	5.24 ± 1.10	0.000
LDL (mmol/L)	2.85 ± 0.83	2.99 ± 0.94	0.000
HDL (mmol/L)	1.37 ± 0.34	1.33 ± 0.33	0.000
Creatinine (μmol/L)	74.97 ± 16.95	78.69 ± 18.02	0.000

Data are expressed as the mean ± standard deviation or n (%), where appropriate. *BMI* body mass index, *SBP* systolic blood pressure, *DBP* diastolic blood pressure, *HDL* high-density lipoprotein, *LDL* low-density lipoprotein, *FBG* fasting blood glucose

**Fig. 3:**
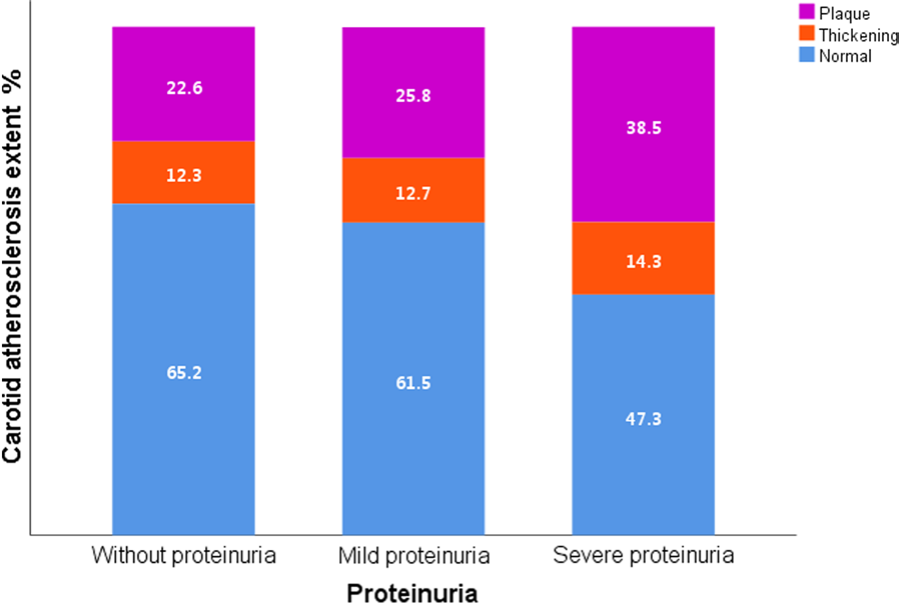
Proteinuria according to the multiterritorial extent of carotid atherosclerosis

The prevalence rate of carotid atherosclerosis was higher in proteinuria populations. Compared with proteinuria-negative subjects, proteinuria-positive subjects had a higher prevalence rate of carotid atherosclerosis. The disparity was statistically significant (22.6% *vs.* 26.7%, χ^2^ = 10.03, p = 0.002) (Table 1). An analysis of subgroups was performed. As shown in the percentage bar diagram between proteinuria level and carotid atherosclerosis level, the carotid atherosclerosis level increased as the proteinuria level increased (as shown in Fig. 3). Their correlation, which was evaluated by the the chi-square test, showed that the linear regression component was statistically significant (χ^2^ = 16.30, p < 0.001). The coefficient of rank correlation was 0.012 (p < 0.001), showing that the carotid atherosclerosis level increased as the proteinuria level increased.

### Multivariate logistic regression

Proteinuria independently correlated with carotid atherosclerosis. After adjusting for factors via multivariate logistic regression, including age, gender, BMI, blood lipids, blood pressure, renal function, hypertension, diabetes mellitus and HLP, proteinuria was found to be an independent risk factor for atherosclerosis. Patients with proteinuria had a higher prevalence rate of atherosclerosis than patients without proteinuria (OR = 1.194, 95% CI 1.018–1.401, p = 0.029) (Table 3). The assignment of the multivariate logistic regression is shown in Table 2.

**Table 2:** Multivariate logistic regression assignment table

Variable name	Variable	Assignment description
X_1_	Proteinuria = Negative	0 = Yes, 1 = No
X_2_	Male	0 = No, 1 = Yes
X_3_	Age (years)	0 = 〝 < 40〞, 1 = 〝 < 50〞, 2 = 〝 < 60〞, 3 = 〝 ≥ 60〞
X_4_	BMI < 24 kg/m^2^	0 = Yes, 1 = No
X_5_	SBP < 130 mmHg	0 = Yes, 1 = No
X_6_	DBP < 90 mmHg	0 = Yes, 1 = No
X_7_	Pulse pressure < 50 mmHg	0 = Yes, 1 = No
X_8_	Hypertension	0 = No, 1 = Yes
X_9_	Diabetes mellitus	0 = No, 1 = Yes
X_10_	Hyperlipidaemia	0 = No, 1 = Yes
X_11_	FBG ≤ 6.1 mmol/L	0 = Yes, 1 = No
X_12_	Triglycerides < 2.3 mmol/L	0 = Yes, 1 = No
X_13_	Total cholesterol < 6.2 mmol/L	0 = Yes, 1 = No
X_14_	LDL < 4.1 mmol/L	0 = Yes, 1 = No
X_15_	HDL < 1.0 mmol/L	0 = Yes, 1 = No
X_16_	Creatinine < 106 μmol/L	0 = Yes, 1 = No
Y	Carotid plaque	0 = Yes, 1 = No

*BMI* body mass index, *SBP* systolic blood pressure, *DBP* diastolic blood pressure, *HDL* high-density lipoprotein, *LDL* low-density lipoprotein, *FBG* fasting blood glucose

According to the principle of multivariate logistic regression, we constructed the following new risk prediction model of carotid atherosclerotic plaque (*P* is the prediction probability) (Table 3):

$$P = \frac{{e^{{ - 4.848 + 0.177x_{1} + 0.861x_{2} + 1.014x_{3} + 0.436x_{5} + 0.282x_{7} + 0.345x_{11} + 0.212x_{12} + 0.326x_{14} }} }}{{1 + e^{{ - 4.848 + 0.177x_{1} + 0.861x_{2} + 1.014x_{3} + 0.436x_{5} + 0.282x_{7} + 0.345x_{11} + 0.212x_{12} + 0.326x_{14} }} }}$$

X_1_ = proteinuria, X_2_ = sex, X_3_ = age, X_5_ = systolic blood pressure, X_7_ = pulse pressure, X_11_ = fasting blood glucose, X_12_ = triglycerides, X_14_ = low-density lipoprotein.

**Table 3:** The results of multivariate logistic regression analysis

Selected variable	β	SE	Wald χ^2^	*p*	Odds ratio (95%CI)
X_1_	0.177	0.081	4.763	0.029	1.194 (1.018–1.401)
X_2_	0.861	0.071	145.973	0.000	2.365 (2.057–2.720)
X_3_	1.014	0.038	696.921	0.000	2.758 (2.558–2.973)
X_5_	0.436	0.086	25.688	0.000	1.547 (1.307–1.831)
X_7_	0.282	0.070	16.331	0.000	1.326 (1.156–1.520)
X_11_	0.345	0.088	15.460	0.000	1.412 (1.189–1.677)
X_12_	0.212	0.076	7.776	0.005	1.237 (1.065–1.436)
X_14_	0.326	0.100	10.549	0.001	1.386 (1.138–1.687)
Constant	− 4.848	0.135	1296.849	0.000	0.008

X_1_ proteinuria, X_2_ sex, X_3_ age, X_5_ systolic blood pressure, X_7_ pulse pressure, X_11_ fasting blood glucose, X_12_ triglycerides, X_14_ low-density lipoprotein

**Fig. 4:**
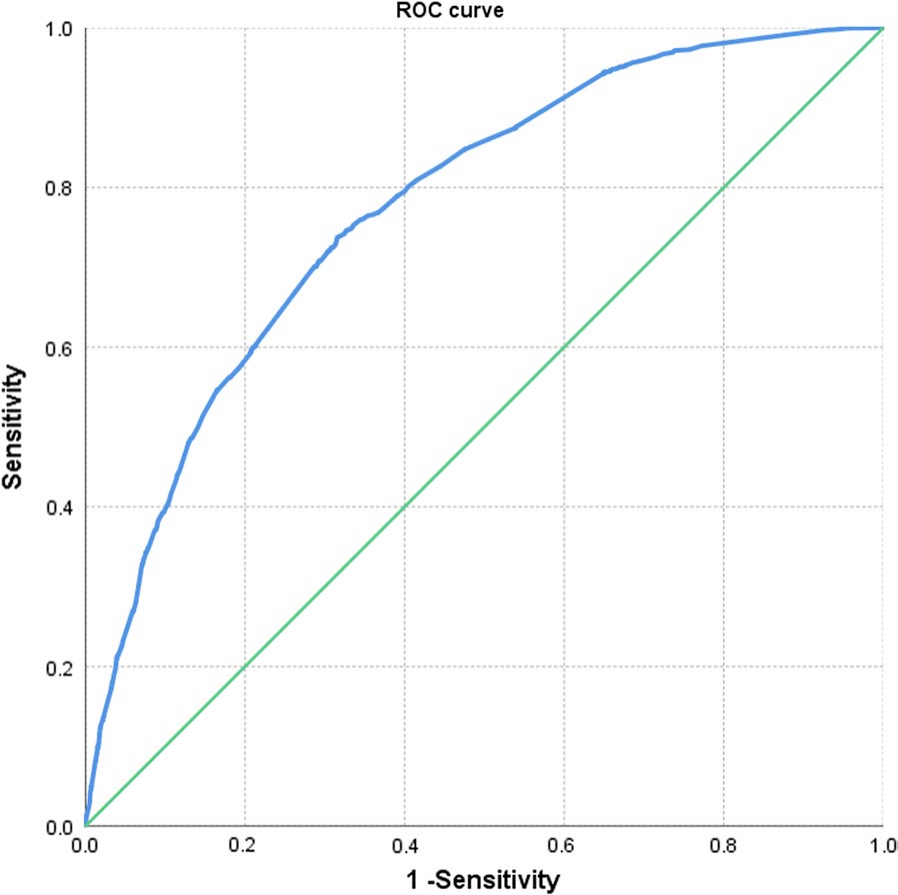
The ROC curve of the newly established multivariate logistic regression model

In our study, the area under the ROC curve of the newly established multivariate logistic regression model for predicting the risk of carotid atherosclerotic plaque in southern China was 0.776 (Fig. 4), and the sensitivity and specificity of the model were 70.8% and 70.6%, respectively. The Hosmer–Lemeshow test was used to test the model, and the results showed that the model has high goodness of fit and strong independent variable prediction ability.

## Discussion

**Fig. 5:**
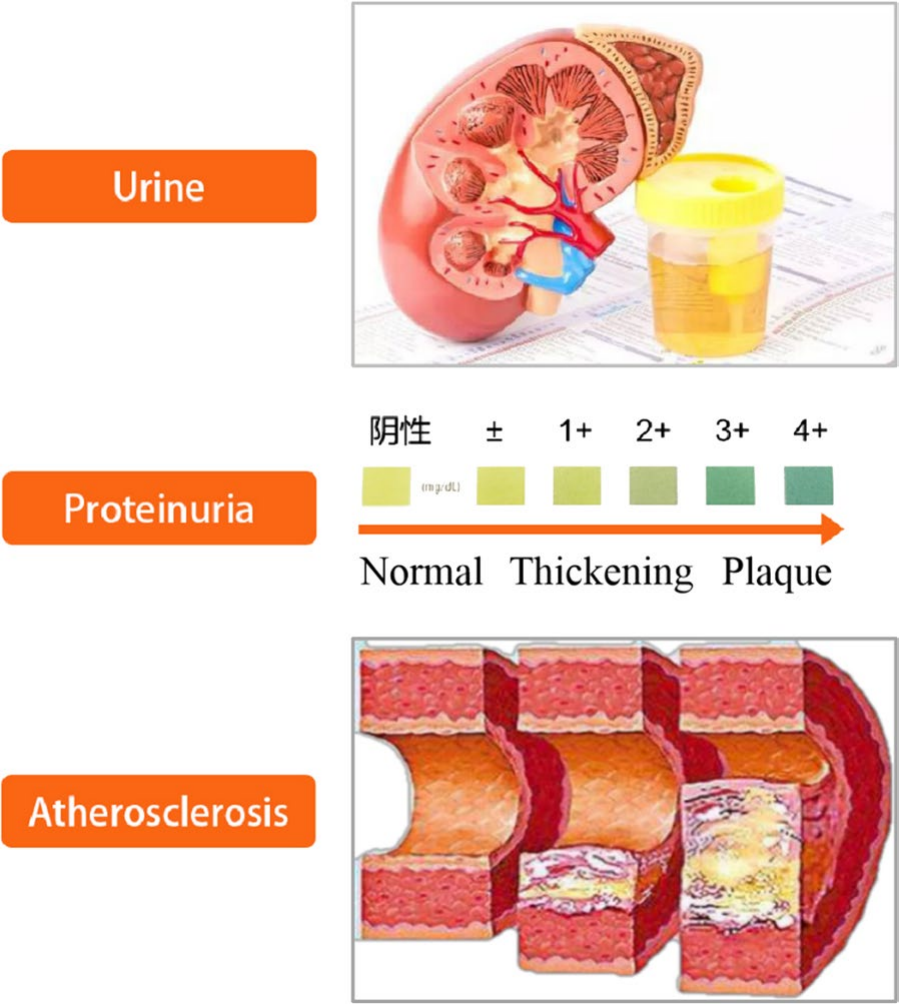
Relationship between urinary protein and atherosclerosis

The study was multicentric, cross-sectional and observational, with general populations as subjects. It aimed to determine the correlation between proteinuria and carotid atherosclerosis in the real world. After adjusting for traditional risk factors, it was found that proteinuria correlated with carotid atherosclerosis. Taking their correlation and risk factors into consideration, the disparity was statistically significant. As the proteinuria level increased, the carotid atherosclerosis level increased (Fig. 5). Thus, proteinuria might be an independent risk factor for atherosclerosis.

Proteinuria has been considered a predictor of renal lesions, and its correlation with atherosclerosis has not been discussed [19]. Traditional risk factors have failed to explain all the disparities in the prevalence rate of atherosclerosis, indicating that studies have to be carried out on new risk factors. New risk factors have been mentioned one after another by scholars, including sleep quality [20], air pollution [21], socioeconomic status [22], Helicobacter pylori infection [23] and visceral adipose tissue volume [24], apart from traditional risk factors, such as age, sex, hypertension, diabetes mellitus, smoking, BMI, HLP, a lack of exercise and too much mental stress. The proteinuria studied in this study might be a new risk factor for atherosclerosis.

Previous studies have mainly focused on the correlation between proteinuria and the mortality of patients with diabetes mellitus or CVD [11–13, 25]. Many classic studies have shown that among patients with diabetes mellitus or CVD, proteinuria is correlated with all-cause mortality. In recent years, Pesola and other scholars have also found that proteinuria could serve as a predictor of all-cause mortality and CVD mortality, which have nothing to do with sex. These studies, however, have not explained how proteinuria affects mortality in a detailed manner or discussed the correlation between proteinuria and atherosclerosis. A hypothesis put forward by Steno showed that proteinuria indicated that blood vessels at the glomerulus level of type 1 diabetic patients have been injured [26], suggesting that atherosclerosis often occurred and that general atherosclerosis increased the occurrence risk of all-cause mortality and CVD mortality. Our study was carried out based on the statistical analysis of large samples, including healthy and subhealthy populations. The finding that proteinuria is correlated with atherosclerosis to some extent verified this hypothesis.

Minyoung and other scholars found that proteinuria was not correlated with atherosclerosis according to a cross-sectional study of 88 type 1 diabetic patients [14]. The result was the opposite, which might be caused by a lack of sufficient samples. All the subjects were type 1 diabetic patients from the same centre. Therefore, there were limitations related to the samples. The subjects in our study were 7405 members of the general population selected from 13,454 patients from multiple centres. The prevalence rate between subjects with proteinuria and subjects without proteinuria differed in the chi-square test, which was statistically significant (22.6% *vs.* 26.7%, χ^2^ = 10.03, *p* = 0.002), and the proteinuria levels had a positive correlation with mortality of atherosclerosis via a linear trend test of subgroups in a bidirectional and orderly way, which showed that the occurrence risk of atherosclerosis increased as the proteinuria level increased (χ^2^ = 16.30, *p* < 0.001), with a coefficient of rank correlation of 0.012 (*p* < 0.001). After adjusting for factors such as age, sex, BMI, blood lipids, blood pressure, renal function, hypertension, diabetes mellitus and hyperlipemia, proteinuria was found to be independently correlated with atherosclerosis (OR = 1.190, 95% CI 1.015–1.397, *p* = 0.033).

Certain former cross-sectional studies with large samples (n = 3312) have shownthat among patients with hypertension or diabetes mellitus, microalbuminuria has something to do with subclinical atherosclerosis [27]. Among patients without clinical CVD, hypertension and diabetes mellitus, microalbuminuria had nothing to do with subclinical atherosclerosis, which was in accordance with our study. Subjects in that study, however, were comparatively older (age ≥ 65), failing to represent the general population. In addition, populations aged above 65 had a higher prevalence rate of atherosclerosis, which would cover proteinuria’s impact on carotid atherosclerosis. In addition, that study failed to include all factors in the regression model, which might have caused errors. Finally, the predictors of that study were microalbuminuria (30–300 μg albumin/mg creatinine) and subclinical atherosclerosis (defined as increased carotid intima-media thickness, decreased ankle-brachial index or increased left ventricular weight). In our study, we used dipstick proteinuria testing and carotid atherosclerosis examination. Therefore, our predictors were comparatively defined. More importantly, our study subjects made up large samples (n = 7405), with the general population aged 18–90. We identified all the possible risk factors related to carotid atherosclerosis to determine the correlation between proteinuria and carotid atherosclerosis.

Most of the risk prediction models of atherosclerosis reported at present are single-centre risk prediction models. The newly established risk prediction model in our study was based on multicentre data and was applicable to a wider range of people. In our study, a new indicator of proteinuria was included, and a risk prediction model for atherosclerosis was established by multifactor logistic regression. Among the 16 clinical risk factors studied, seven factors were included in the model, including proteinuria, age, sex, pulse pressure, hypertension, triglycerides and low-density lipoprotein, which are common and easily accessible indicators. Theoretically, the atherosclerosis risk prediction model established in our study was more applicable than the models established in other single-centre studies.

Although our study has a multicenter design, all the 4 centers were located in a limited geographic area (Southern China). However, the prevalence of dyslipidemia and other risk factors in this Asian population was similar to that reported in large contemporary trials and real-world registries including other ethnicities. The former assessed the relationship between different antiplatelet therapy regimens and patients’ characteristics [28]. The latter described recent demographics and therapeutic changes in the Italian acute coronary syndromes population [29]. This was relevant and potentially supports the generalizability of the our results to patients of other regions/countries.

Some indices in routine examination may also have certain value in the prediction and prognosis of cardiovascular disease. Recent studies have found that haemoglobin is independently associated with mortality from CVD. Leonardi and his team used multivariate Cox regression to find that among patients with ACS managed invasively, an in-hospital haemoglobin drop ≥ 3 g/dl, even in the absence of overt bleeding, is common and is independently associated with an increased risk of 1-year mortality [30]. In addition, the Academic Research Consortium (ARC) for High Bleeding Risk (HBR) proposed clinical and biochemical criteria for High Bleeding Risk (HBR) for the identification of HBR patients. After verification, it was found that all major and the majority of the minor ARC-HBR criteria were identified in isolation patients at HBR [31].

Our study had obvious advantages. First, this was the first study on the correlation between dipstick proteinuria testing and the risk of carotid atherosclerosis. Second, the study was a multicentre study with members of the general population in large samples, which strengthened the reliability of the correlation between proteinuria and carotid atherosclerosis. Third, the predictors in this study were absolutely defined, such as proteinuria “negative, ± ,1 + , ≥ 2 + ” and carotid atherosclerosis. Although dipstick proteinuria testing had certain chance factors, proteinuria was still positively correlated with carotid atherosclerosis, which strengthened the reliability of the evidence. Fourth, the correlation between proteinuria and atherosclerosis provided a cost-effective screening predictor for CVD prevention from death to atherosclerosis.

The study also had certain limitations. First, compared with cohort studies, the causal relationship provided by cross-sectional studies was comparatively weak. Second, a history of smoking was not included in our study data, which might have affected the accuracy of the results. In the first stage of the study, a history of smoking did not serve as a factor because Chinese people were not clear about definitions of smoking, secondhand smoking, public areas and so on [32]. Third, the optimal urine test was to collect 24-h urine, which was hard to carry out. Therefore, the advantage was converted into a disadvantage. Although the revealed and unrevealed groups had approximate values, it was found that there was a disparity in the prevalence rate of carotid atherosclerosis, which was undoubtedly and obviously underestimated. Finally, proteinuria increased the occurrence risk of atherosclerosis, which needs to be confirmed by further study. Our study could provide certain evidence to some extent.

Based on the discovery from our study, the screening of the risk factor proteinuria promotes CVD prevention of death due to atherosclerosis, especially during the period of COVID-19. Currently, CVD brings increasing burdens to the whole medical system. In addition, during the period of COVID-19, patients with CVD are especially weak [33, 34]. Although proteinuria has been proven to be related to all-cause mortality, focus on death cannot effectively help CVD prevention. Attention must be paid to risk factors for CVD, which can lower the prevalence rate of CVD and reduce social and medical burdens [35]. In our study, proteinuria was found to be a risk factor for atherosclerosis, and dipstick proteinuria testing was cost-effective and could serve as the optimal method of disease testing and treatment in undeveloped countries. Especially during the period of COVID-19, patients only have to send their urine samples to the hospital, which is the biggest advantage of this method compared with other high-cost testing methods in other medical institutions.

## Conclusions

As mentioned above, our study shows that proteinuria independently correlates with atherosclerosis. Taking their correlation and risk factors into consideration, the disparity is statistically significant. As the proteinuria level increases, the atherosclerosis level increases. Based on the results of the study, it is advisable to pay much attention to atherosclerosis screening in patients to promote CVD prevention. From a public health standpoint, our study shows that dipsticks are cost-effective and time-saving (the testing time is within 1 min), which can be the optimal method of disease diagnosis and treatment. Even in the period of COVID-19, it plays a role in screening out the risk factors for CVD. In this study, the newly established multivariate logistic regression model for predicting the risk of atherosclerosis had a high goodness of fit and strong ability to predict independent variables, so it has a certain clinical value.

## Data Availability

Email *jiangwencai666@163.com* and the data and materials can be accessed.
